# Leukodepleted Packed Red Blood Cells Transfusion in Patients Undergoing Major Cardiovascular Surgical Procedure: Systematic Review and Meta-Analysis

**DOI:** 10.1155/2019/7543917

**Published:** 2019-02-25

**Authors:** Daniel Simancas-Racines, Ingrid Arevalo-Rodriguez, Gerard Urrutia, Diana Buitrago-Garcia, Solange Núñez-González, María José Martínez-Zapata, Eva Madrid, Xavier Bonfill, Ricardo Hidalgo-Ottolenghi

**Affiliations:** ^1^Cochrane Ecuador, Centro de Investigación en Salud Pública y Epidemiología Clínica (CISPEC), Facultad de Ciencias de la Salud Eugenio Espejo, Universidad UTE, Quito 170129, Ecuador; ^2^Clinical Biostatistics Unit, Hospital Universitario Ramon y Cajal (IRYCIS), CIBER Epidemiology and Public Health (CIBERESP), Madrid 28034, Spain; ^3^CIBER Epidemiología y Salud Pública (CIBERESP), Iberoamerican Cochrane Centre Biomedical Research Institute Sant Pau (IIB Sant Pau), Barcelona 08041, Spain; ^4^Centro Interdisciplinario de Estudios en Salud (CIESAL), Escuela de Medicina, Universidad de Valparaíso, Cochrane Chile, Valparaíso 2391415, Chile

## Abstract

**Background:**

Leukocytes contained in the allogeneic packed red blood cell (PRBC) are the cause of certain adverse reactions associated with blood transfusion. Leukoreduction consists of eliminating leukocytes in all blood products below the established safety levels for any patient type. In this systematic review, we appraise the clinical effectiveness of allogeneic leukodepleted (LD) PRBC transfusion for preventing infections and death in patients undergoing major cardiovascular surgical procedures.

**Methods:**

We searched randomized controlled trials (RCT), enrolling patients undergoing a major cardiovascular surgical procedure and transfused with LD-PRBC. Data were extracted, and risk of bias was assessed according to Cochrane guidelines. In addition, trial sequential analysis (TSA) was used to assess the need of conducting additional trials. Quality of the evidence was assessed using the GRADE approach.

**Results:**

Seven studies met the eligibility criteria. Quality of the evidence was rated as moderate for both outcomes. The risk ratio for death from any cause comparing the LD-PRBC versus non-LD-PRBC group was 0.69 (CI 95% = 0.53 to 0.90; *I* ^2^ = 0%). The risk ratio for infection in the same comparison groups was 0.77 (CI 95% = 0.66 to 0.91; *I* ^2^ = 0%). TSA showed a conclusive result in this outcome.

**Conclusions:**

We found evidence that supports the routine use of leukodepletion in patients undergoing a major cardiovascular surgical procedure requiring PRBC transfusion to prevent death and infection. In the case of infection, the evidence should be considered sufficient and conclusive and hence indicated that further trials would not be required.

## 1. Introduction

Blood transfusion is an acute intervention implemented to solve life- and health-threatening conditions on a short-term basis [1]. Packet red blood cell (PRBC), prepared by removing plasma from whole blood, is typically used to transfuse anemia patients who require infusion of red blood cell (RBC) to restore tissue oxygenation. However, PRBC transfusion is associated with an increasing risk of infectious and noninfectious adverse events, the most common among which are nonhemolytic febrile transfusion reactions, human leukocyte antigen (HLA) alloimmunization and platelet refractoriness observed in multitransfused patients, and transmission of leukotropic viruses. One of the strategies commonly used to prevent posttransfusion complications is leukodepletion, that is, a process by which the white blood cells (WBCs) are intentionally reduced by almost 99.99% to PRBC. According to the current standards, PRBC residual leukocytes require to be <5 × 10^6^ cells per unit according to the FDA or <1 × 10^6^ cells per unit according to the Council of Europe [2].

Several studies have focused on the advantages of leukodepleted PRBC for transfusion in cardiac surgery [3–8], colorectal surgery [4, 9–13], gastrointestinal surgery [4, 7, 14], and renal transplantation [15–17]. Cardiac surgery accounts for a large proportion of the blood transfusions administered each year. Transfusion rates have been reported from 7.8% to 92.8% for combination of coronary artery bypass graft (CABG) surgery with valve or other major surgical interventions [18].

Although blood transfusions are necessary in major cardiovascular surgery, several studies found that blood transfusions had also deleterious effects. Considering the abovementioned reasons, this subgroup of the surgical population is of special interest for the analysis of the effectiveness and safety of transfusion practices. Several studies have been showed that cardiac surgery is related with tissue trauma, ischemia-reperfusion injury, and blood surface contact. These clinical settings induce systemic effects and release of inflammatory mediators, which are supposed to play a role in the development of systemic inflammatory response syndrome (SIRS), multiple organ dysfunction syndrome (MODS), infections, and postoperative complications [19, 20]. Additionally, in cardiac surgery, PRBC are frequently transfused and these transfusions have been found to be associated (dose-dependent) with an increased risk of postoperative infections and mortality after cardiac surgery [21–25]. It is not clear what the possible mechanisms that clarify this association could be [26] although the presence of allogeneic leukocytes in PRBC are hypothetical to play a fundamental role, probably by evolving into the inflammatory response after cardiac surgery. In support of this reasoning, Bilgin et al. found higher concentrations of proinflammatory mediators (such as IL-6 and IL-10) during the postoperative period in cardiac valve surgery patients receiving allogeneic leukocyte-containing blood transfusions compared with leukocyte-depleted blood transfusions [27]. This, in turn, would support the potential benefits of the routine use of leukodepleted PRBC transfusion in the setting of cardiac surgery to reduce infectious complications [28].

One previous review reported that there is no clear evidence supporting the effectiveness of leukodepleted PRBC for preventing transfusion-related acute lung injury (TRALI) or reducing mortality and infectious or noninfectious complications in patients undergoing any type of surgery [29]. However, a considerable heterogeneity in the pooled estimation was found due to the inclusion of different types of populations (oncology, trauma, and cardiac surgery patients) which may have prevented the detection of beneficial effects in some particularly relevant subgroups of surgical patients. Therefore, the objective of this review was to assess the effects of LD-PRBC in patients undergoing major cardiovascular surgical procedure, who are more likely to suffer significant blood loss [20] and consequently have a much higher probability to receive transfusions of blood products [18].

## 2. Methods

We conducted a systematic review of randomized clinical trials (RCTs). The protocol was registered in PROSPERO, an international prospective register of systematic review protocols (registration number: CRD42018103104).

### 2.1. Inclusion and Exclusion Criteria

To be included in the review, studies had to meet the following criteria: randomized controlled trial conducted with patients of any age undergoing a major cardiovascular surgical procedure (such as valve surgery, cardiac bypass, and aneurysm repair), requiring allogeneic PRBC, with the aim of comparing LD-PRBCs versus non-LD-PRBCs. Besides, studies had to report results on death from any cause and infection from any cause (unspecified). We excluded studies with other designs or that included patients transfused with other blood components as a principal intervention.

### 2.2. Search Strategy

We carried on sensitive electronic searches in the Cochrane Injuries Group Specialized Register, the Cochrane Central Register of Controlled Trials (CENTRAL, the Cochrane Library), MEDLINE (OVID 1946 to present), EMBASE (Elsevier), LILACS, Clinical Trials register (http://www.clinicaltrials.gov), and the WHO International Clinical Trials Registry Platform (http://apps.who.int/trialsearch/). We ran the most recent search on June 10^th^, 2018 (see Supplementary material (available here) for details).

### 2.3. Screening, Data Extraction, and Assessment of Risk of Bias

Two review authors independently screened all titles and abstracts retrieved by the search against the selection criteria and obtained full texts when necessary. All decisions regarding inclusion and exclusion were made by consensus. Data extraction was performed in duplicate and risk of bias (RoB) assessment of the included studies, following the domain-based evaluation method described in the Cochrane Handbook for Systematic Reviews of Interventions [30].

As a support to establish our conclusions on the effects of leukodepleted PRBC, we developed a “Summary of Findings” table using the GRADE approach for assessing the quality of evidence, according to the methods and recommendations described in the Cochrane Handbook for Systematic Reviews of Interventions [30].

### 2.4. Statistical Analysis

We calculated the treatment pooled effect for death from any cause and infection from any cause by means of the risk ratio (RR) with the corresponding 95% confidence intervals (CI), using the random-effects model approach for data pooling in the meta-analysis, which accounts for statistical heterogeneity across studies and leads to a more conservative estimate of the effect. We estimated the statistical heterogeneity in the meta-analysis by using the *I* ^2^ statistics [31]. All these analyses were carried out using RevMan 5.3 [32].

We used trial sequential analyses (TSA) to estimate the required information size for death from any cause and infection from any cause in order to reduce the risk of random errors in our conclusions and calculating the required information size for a meta-analysis. This analysis makes available an adjusted statistical threshold for benefits, harms, or futility before the required information size was reached [33, 34]. By using this method, we aimed at controlling the risk of type I and type II errors due to sparse data and repetitive testing of accumulating data [33, 35–37].

## 3. Results

### 3.1. Literature Search Results

**Figure 1:**
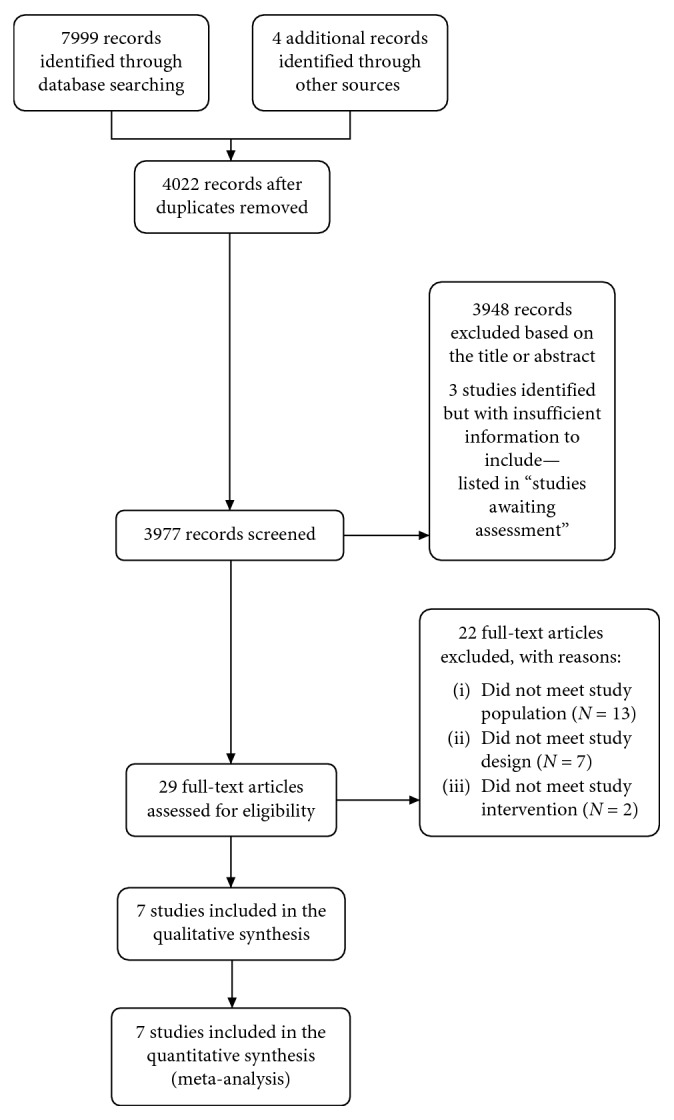
Flow diagram of the literature search and study selection.

We initially identified 7,999 records from the search strategies updated until June 2018 [23] and four more from other sources. After removing duplicates, 4,022 were manually screened, and 3,993 records were excluded for title and abstract. We reviewed the full text of 29 studies, 22 of which were excluded. Finally, only seven RCTs with 3,154 participants were included into the qualitative and quantitative analysis of this report [3, 5–8, 38, 39] (Figure 1).

### 3.2. Characteristics of the Included Studies

Two of the seven included studies only had abstract available [38, 39]. Three studies were carried out in the Netherlands (60%) [3, 6, 7]. All studies included adult patients, with mean ages greater than 60 years. Leukodepletion process was described only in three of the seven studies, using three different criteria (1.2 ± 1.4 × 10^6^, 5 × 10^6^, or 0.15 ± 0.02 × 10^6^, leukocytes per unit) [3, 5, 6].

Sample size for the transfused patients ranged from 38 to 304 (mean 189) for the leukodepleted group and 31 to 303 (mean 207) for the comparator group. In van Hilten 2004 study, we included only patients undergoing aneurysm repair, excluding gastrointestinal oncology surgery. From the van de Watering 1998 study, we included the stored-filtered (SF) group within the leukodepleted group and the packed cells (PC) group within the comparator group.  Table 1 describes the main characteristics of included studies. Regarding risk of bias, most studies were assessed as “unclear risk” regarding selection bias (random sequence generation and allocation concealment) due to lack of details in the study report. Only one study was assessed as having “low risk” of bias for blinding of participants, personnel, and outcome assessors [6]. We considered the missing outcome data shown in van Hilten 2004 as having a “high risk” of bias, due to fact that losses are likely to be related to the main outcomes [7]. In addition, three studies were considered as at “unclear risk” of other bias [3, 7, 8, 38, 39].

### 3.3. Target Death from Any Cause

**Figure 2:**
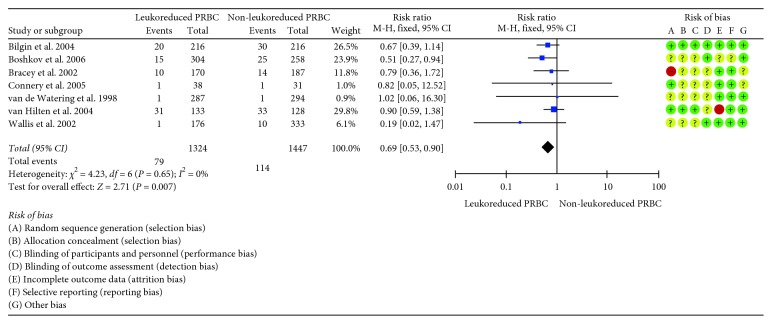
Forest plot of included studies evaluating LD-PRBC versus non-LD-PRBC in patients undergoing a major cardiovascular surgical procedure: death from any cause outcome.

In the included studies, death was assessed at 30 days [8], 60 days [3], 90 days [5, 6], and up to twelve months [38]. Two studies did not report the follow-up time for the death from any cause outcome [7, 39]. The overall death from any cause at the last follow-up was 5.96% (79 events). The pooled RR for the comparison of LD-PRBC versus non-LD-PRBC was 0.69 (CI 95% = 0.53 to 0.90; *I* ^2^ = 0%), thus showing a statistically significant reduction in the risk of death from any cause with LD-PRBS (31% relative reduction) (Figure 2).

**Figure 3:**
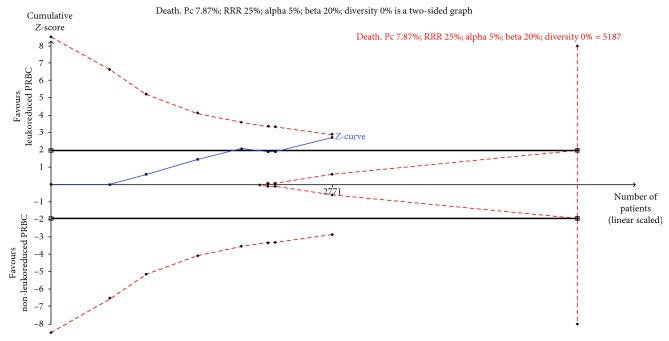
TSA calculated to reliably detect a 25% relative change in the incidence of death from any cause, assuming a control group event rate of 8.99% with a power of 80% at an alpha of 5%. Notes: DARIS: diversity adjusted required information size; Pc: event proportion in the control group; RRR: relative risk reduction in the intervention group; (a) type I error; (b) type II error; DIVERSITY: diversity (D-square). Dead: the required information size was 5,187 participants. The cumulative *Z*-score (blue line) did not cross the trial sequential monitoring boundaries for benefit (red lighter inward sloping line) after the seven trials.

We conducted TSA analysis to determine the reliability of one of the outcomes of this systematic review: death from any cause (Figure 3). TSA of LD-PRBC compared with control non-LD-PRBC indicated that the optimal information size needed to reliably detect a plausible effect was 5,187 patients. However, 2,771 (53.4%) patients had so far been collected. The cumulative *z*-curve of all trials crossed the traditional boundary but did not cross the trial sequential monitoring boundary. The TSA *α*-spending adjusted 95% CI overlapped with no effect (RR 0.49 and RR 1.02, respectively); thus, the TSA yielded an inconclusive result about the true effect of LD-PRBC in preventing death from any cause. Therefore, for death from any cause outcome, more RCTs are needed (Figure 3). According to GRADE criteria, the quality of the evidence was moderate to low (Table 2).

### 3.4. Target Infection

**Figure 4:**
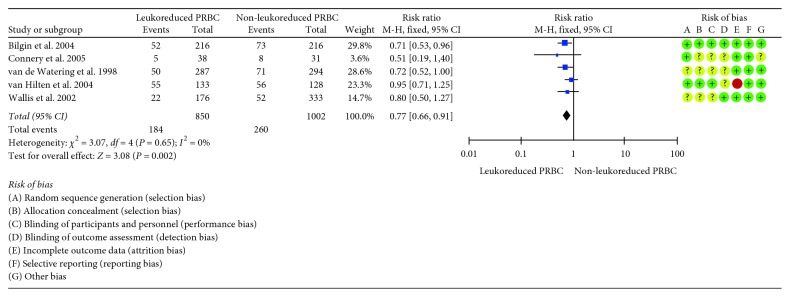
Forest plot of included studies evaluating LD-PRBC versus non-LD-PRBC in patients undergoing a major cardiovascular surgical procedure: infection outcome.

Regarding infection, five out of seven included studies reported this outcome [3, 5–8, 38, 39]. Incidence of infection after follow-up was 19.8% (494 events). The pooled RR for the comparison of the LD-PRBC versus non-LD-PRBC group was 0.77 (IC 95% = 0.66 a 0.91; *I* ^2^ = 0%), thus showing a statistically significant reduction in the risk of infection with LD-PRBS (23% relative risk reduction) (Figure 4).

**Figure 5:**
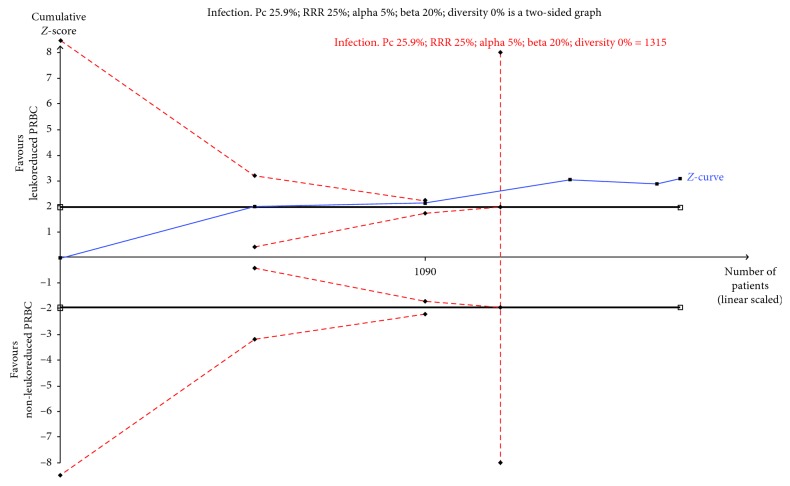
TSA calculated to reliably detect a 25% relative change in the incidence of infection from any cause, assuming a control group event rate of 24.6% with a power of 80% at an alpha of 5%. Notes: DARIS: diversity adjusted required information size; Pc: event proportion in the control group; RRR: relative risk reduction in the intervention group; (a) type I error; (b) type II error; DIVERSITY: diversity (D-square). Infection: the required information size was 1,315 participants. The cumulative *Z*-score (blue line) crossed the trial sequential monitoring boundaries for benefit (red lighter inward sloping line) after the second trial (1,090 participants); thus, the risk of random error in the finding can be excluded. Therefore, it is not necessarily additional testing based on the assumed intervention effect of the RRR of 25%, an alpha of 5%, and a beta of 20% with respect to this result.

We conducted TSA to determine the reliability of one of the outcomes of this systematic review: infection from any cause. TSA of LD-PRBC compared with non-LD-PRBC indicated that the optimal information size needed to reliably detect a plausible effect was 1,315 patients. However, the accumulate data of 1,852 participants constituted more than 100% of the optimal information size calculated. The cumulative *z*-curve of all trials crossed the traditional boundary as well as the trial sequential monitoring boundary. The TSA *α*-spending adjusted 95% CI, did not overlap the zone of no effect (RR 1.0), and is compatible with a potential benefit (RR 0.65 and RR 0.93, respectively); thus, the TSA yielded a conclusive result about the true effect of LD-PRBC in preventing infection from any cause. Therefore, for infection from any cause outcome, no more RCTs are needed (Figure 5). According to GRADE criteria, the quality of the evidence was moderate (Table 2).

## 4. Discussion

In this systematic review, we showed that patients undergoing a major cardiovascular surgery who were transfused with LD-PRBCs might benefit from a decreased risk of infections and death from any cause. The certainty for the first outcome is moderate according to the quality of the body of evidence available, but conclusive according to the TSA analysis. As for the later outcome, the certainty in the result is also moderate but not as conclusive as regarding the former result.

Several reviews and meta-analyses on postoperative infection and death related to the leukoreduction of blood products have been carried out previously [29]. However, as far as we know, no systematic reviews or meta-analysis with TSA focused specifically in patients undergoing a major cardiovascular surgery have yet been published. Nevertheless, it is important to point out that numerous studies have sought to demonstrate the benefit of leukocytes reduction from red blood cell concentrates in different scenarios.

One previously published Cochrane systematic review comparing LD-PRBC with non-LD-PRBC in all type of surgical patients requiring transfusion, showed a non-significant decrease in the risk of infection (10 trials with 6,709 patients) and all-cause mortality (9 trials with 6,485 patients). However, these results were limited by a significant heterogeneity [29]. These findings contradict our results partially because the population included in that review was very heterogeneous which may have hidden the effect of the intervention in specific subgroups of interest. In contrast, our study was specifically focused in patients undergoing major cardiovascular surgery. This surgery has been related to a higher volume of PRBC transfused per patient compared to colorectal and gastrointestinal surgery, as well as the fact that the leukocytes are transfused to an already activated inflammatory system caused by cardiopulmonary bypass [40]. Thus, it is reasonable to assume that the potential harms of using non-LD-PRBC are higher than in other surgical scenarios.

In contrast, the findings of other systematic reviews are in accordance with our results. One systematic review showed a 50% reduction in the risk of a postoperative infection [41], and another one analyzing “only patients who received transfusion” found a statistically signiﬁcant reduction of 40% in postoperative infection risk, but a nonsigniﬁcant reduction on mortality [42]. However, these two reviews have several limitations. They included studies that used other blood components apart from LD-PRBC cells as an intervention, as well as nonrandomized studies. Moreover, heterogeneity between studies was not taken into account, and the risk of bias of included studies was not appropriately assessed. Furthermore, some relevant studies were not included, and patients undergoing major cardiovascular surgery were not evaluated independently. Finally, any of the aforementioned reviews did not perform a trial sequential analysis, in order to control the risks of type I and type II errors due to sparse data and repetitive testing of accumulating data in all of calculated meta-analyses.

Applicability of this evidence to daily clinical practice is restricted for several reasons. Firstly, external validity may be limited to patients undergoing the same major cardiovascular surgery procedures that have been included in this review. Secondly, the identified studies did not adequately report several factors related to the transfusion of RBC practices that need to be considered when interpreting the results, such as the use of LD or non-LD platelets as a cointervention, the timing of LD (pre-post-storage), and the type of the filter used, among other factors. Thirdly, the number of units transfused in major cardiovascular surgery is massive in most of the cases compared with other surgical and nonsurgical transfusion clinical settings, and therefore, patients undergoing major cardiovascular surgery may suffer a posttransfusion complication is more likely. Finally, the studies reported different definitions for infections, and the mortality was assessed in different time periods (30 days, 60 days, 90 days, and up to twelve months).

Most developed countries currently recommend universal LD-PRBC. However, high costs associated with this procedure, such as the direct costs of LD-PRBC and other associated costs (i.e., costs associated with maintaining dual inventories of leukodepleted PRBC and non-leukodepleted PRBC), merit special attention [43]. As a main strength of this report, we applied Cochrane systematic review methodology throughout all the process. However, despite our effort to include all published studies evaluating LD-PRBC for the prevention of infection and death from any cause in patients undergoing major cardiovascular surgery, it is possible that not all studies were identified. The Bilgin 2004 and Connery 2005 studies reported the use of platelets as a cointervention, which could intervene as a confounder in the analyses. The study Kremke et al. concluded that platelet transfusion of CABG is not associated with increased postoperative mortality [44]; on the contrary, the study Mangano observed a strong relationship between perioperative platelet transfusion and increased postoperative mortality [45]. The effect of platelets on major cardiovascular surgery is not yet clear; however, we have decided to include the studies with the use of platelets due to their common practice, and we recommend analyzing this variable in future studies. The small number of trials identified in our review raises concern about publication bias. However, we demonstrated by means of the TSA analysis that no additional RCTs need to be conducted in order to demonstrate the beneficial effects in terms of preventing infection complications.

## 5. Conclusions

There is clear evidence for supporting the routine use of leukoreduction in patients undergoing a major cardiovascular surgical procedure for preventing infection from any cause. Based on TSA analysis, it is not necessary to conduct more RCTs to assess the effects on infection complications risk reduction. The quality of the evidence is moderate for this outcome and therefore the certainty as well. As for death from any cause, a beneficial effect of LD-PRBC in patients undergoing a major cardiovascular surgical procedure was also observed, but more RCTs are needed to confirm our findings. More research could be justified specifically in those middle and low incomes countries in which LD-PRBC has not been implemented universally yet and/or where the costs of the procedure could be a barrier.

## Figures and Tables

**Table 1:** Characteristics of the included studies.

ID	Country	Age mean by group (LD, C)^*∗*^	Male (%)	Leukodepleted group				Comparator group		Cointerventions
				Transfused patients,*n*	Type of surgery	Leukodepleted definition	Filter	Transfused patients, *n*	Comparator	
Bilgin et al. 2004 [6]	Netherlands	65.3, 66.6	53 to 57	216	Cardiac valve surgery with or without coronary artery bypass graft	0.15 ± 0.02 × 10^6^ per unit	Cellselect-Optima	216	Buffy coat depleted packed cells	Platelets
Boshkov et al. 2006 [38]	USA	Unclear	Unclear	304	Coronary artery bypass graft and/or cardiac valve replacement	Unclear	Unclear	258	Standard RBC	No
Bracey et al. 2002 [39]	USA	Unclear	Unclear	170	Open-heart surgery, coronary artery bypass graft, and valve replacement	Unclear	Unclear	187	Standard RBC	Unclear
Connery et al. 2005 [8]	USA	62.9, 66	71 to 74.2	38	Coronary artery bypass graft	Unclear	Unclear	31	Standard RBC	Platelets
van de Watering et al. 1998 [3]	Netherlands	62.9, 64.4	72.2 to 73.7	287	Coronary artery bypass graft and/or cardiac valve surgery	1.2 ± 1.4 × 10^6^ per unit	Cellselect-optima	294	Buffy coat depleted packed cells	No
van Hilten et al. 2004 [7]	Netherlands	66, 71	Unclear	133	Acute aneurysm surgery and elective aneurysm surgery	Unclear	Unclear	128	Buffy coat depleted packed cells	No
Wallis et al. 2002 [5]	UK	61.7, 62.4	Ratio men/women: 2.6 to 2.9	176	Coronary artery bypass graft and/or cardiac valve surgery	5 × 10^6^ per unit	BPF4	333	Buffy coat depleted packed cells and red blood cells concentrate with plasma reduction	No

RBCs = red blood cells. ^*∗*^LD: leukodepleted group; C: comparator group.

**Table 2:** Summary of findings: GRADE criteria.

Leukodepleted packed red blood cells transfusion in patients undergoing a major cardiovascular surgical procedure						
*Patient or population*: patients undergoing a major cardiovascular surgical procedure transfused with allogeneic packed red blood cells (PRBC)						
*Setting*: hospital						
*Intervention*: leukodepleted (PRBC)						
*Comparison*: non-leukodepleted (PRBC)						

Outcomes	Anticipated absolute effects^*∗*^ (95% CI)		Relative effect (95% CI)	No. of participants (studies)	Certainty of the evidence (grade)	Comments
	Risk with non-leukodepleted (PRBC)	Risk with leukodepleted (PRBC)				

*Death*. Number of events of the total number of transfused patients reported	79 per 1.000	**54 per 1.000** (42 to 71)	**RR 0.69** (0.53 to 0.90)	2771 (7 RCTs)	⊕⊕⊕◯ Moderate^a,b^	TSA yielded an inconclusive result.

*Infection*. Number of events of the total number of transfused patients reported	259 per 1.000	**200 per 1.000** (171 to 236)	**RR 0.77** (0.66 to 0.91)	1852 (5 RCTs)	⊕⊕⊕◯ Moderate^a,b^	TSA yielded a conclusive result.

^*∗*^The risk in the intervention group (and its 95% confidence interval) is based on the assumed risk in the comparison group and the relative effect of the intervention (and its 95% CI). CI: confidence interval; RR: risk ratio. The overall certainty in the evidence should be assessed for each important outcome using four or three categories (such as high, moderate, low, and/or very low) and definitions for each category that are consistent with the definitions used by the GRADE Working Group. ^a^Downgraded because one study has high risk of bias due to attrition bias; another study has other risk of bias at high risk of bias; three studies have unclear risk of bias in generation and allocation concealment of random sequence. ^b^Downgraded due to high risk of bias; one study has high risk of bias due to attrition bias; another study has high risk of bias in a random sequence; five studies have unclear risk of bias in generation and allocation concealment of random sequence.

## Supplementary Materials

Supplementary Materials Appendix 1: detailed search strategy.
