# Supplementary material for: Leukodepleted Packed Red Blood Cells Transfusion in Patients Undergoing Major Cardiovascular Surgical Procedure: Systematic Review and Meta-Analysis
Source: Cardiol Res Pract. 2019 Feb 25;2019:7543917. doi: 10.1155/2019/7543917 (PMC6410443; doi:10.1155/2019/7543917)
Supplement: Supplementary Materials — Appendix 1: detailed search strategy. [file 7543917.f1.docx]

**Appendix 1. Search strategies**

**Cochrane Injuries Group Specialised Register**

“blood transfusion” AND (leuk* OR leuc* OR plasmapheresis OR cytapheres OR apheresis)

**Cochrane Central Register of Controlled Trials (CENTRAL, the Cochrane Library)**

#1MeSH descriptor Blood Component Removal explode all trees

#2MeSH descriptor Leukocyte Reduction Procedures explode all trees

#3MeSH descriptor Cytapheresis explode all trees

#4(plasmapheresis or cytapheres* or apheresis or plateletpheresis or pheresis or phereses or aphereses or leukapheresis or leucapheresis)

#5(Leukoreduc* or leukodeplet* or leukoﬁlt* or leukocyte-reduc* or leucoreduc* or leucodeplet* or leucoﬁlt* or desleucotizat*)

#6buffy coat-depleted

#7leukocyte count or leukocyte free or leucocyte count or leucocyte free

#8((Blood or white blood cell* or WBC or plasma) NEAR/3 (reduc* or deplet* or replete* or remov* or ﬁltrat* or ﬁlter* or cytapheresis))

#9((leukocyte* or leucocyte*) NEAR/3 (reduc* or deplet* or replete* or remov* or ﬁltrat* or ﬁlter*))

#10(#1 OR #2 OR #3 OR #4 OR #5 OR #6 OR #7 OR #8 OR #9)

#11MeSH descriptor Blood Transfusion explode all trees

#12((allogenic or allogeneic) NEAR/3 blood transfusion*)

#13(blood component* NEAR/3 transfusion*)

#14((erythrocyte* or leukocyte* or platelet* or RBC or red blood cell* or WBC or white blood cell* or thrombocyte* or blood) NEAR/

3 Transfusion*)

#15(#11 OR #12 OR #13 OR #14)

#16(#10 AND#15)

**Medline (OvidSP)**

1.exp Blood Component Removal/

2.exp Leukocyte Reduction Procedures/

3.exp cytapheresis/

4.(plasmapheresis or cytapheres* or apheresis or plateletpheresis or pheresis or phereses or aphereses or leukapheresis or leucapheresis).ab,ti.

5.(Leukoreduc*

or leukodeplet* or leukoﬁlt* or leukocyte-reduc* or leucoreduc* or leucodeplet* or leucoﬁlt* or desleucotizat*).mp.

6.buffy coat-depleted.ab,ti.

7.(leukocyte count or leukocyte free or leucocyte count or leucocyte free).ab,ti.

8.((Blood or white blood cell* or WBC or plasma) adj3 (reduc* or deplet* or replete* or remov* or ﬁltrat* or ﬁlter* or cytapheresis)).ab,ti.

9.((leukocyte* or leucocyte*) adj3 (reduc* or deplet* or replete* or remov* or ﬁltrat* or ﬁlter*)).ab,ti.

10.or/1-9

11.exp Blood Transfusion/

12.((allogenic or allogeneic) adj3 blood transfusion*).ab,ti.

13.(blood component* adj3 transfusion*).ab,ti.

14.((erythrocyte* or leukocyte* or platelet* or RBC or red blood cell* or WBC or white blood cell* or thrombocyte* or blood) adj3

Transfusion*).ab,ti.

15.or/11-14

16.10 and 15

17.randomi?ed.ab,ti.

18.randomized controlled trial.pt.

19.controlled clinical trial.pt.

20.placebo.ab.

21.clinical trials as topic.sh.

22.randomly.ab.

23.trial.ti.
24.17 or 18 or 19 or 20 or 21 or 22 or 23
25.(animals not (humans and animals)).sh.
26.24 not 25
27.26 and 16

**Embase + Embase Classic (OvidSP)**

1. exp Blood Component Removal/

2. exp Leukocyte Reduction Procedures/

3. exp cytapheresis/

4. (plasmapheresis or cytapheres* or apheresis or plateletpheresis or pheresis or phereses or aphereses or leukapheresis or leucapheresis).ti,ab.

5. (Leukoreduc* or leukodeplet* or leukoﬁlt* or leukocyte-reduc* or leucoreduc* or leucodeplet* or leucoﬁlt* or desleucotizat*).ti,ab.

6. buffy coat-depleted.ti,ab.

7. (leukocyte count or leukocyte free or leucocyte count or leucocyte free).ti,ab.

8. ((Blood or white blood cell* or WBC or plasma) adj3 (reduc* or deplet* or replete* or remov* or ﬁltrat* or ﬁlter* or cytapheresis)).ti,ab.

9. ((leukocyte* or leucocyte*) adj3 (reduc* or deplet* or replete* or remov* or ﬁltrat* or ﬁlter*)).ti,ab.

10. 1 or 2 or 3 or 4 or 5 or 6 or 7 or 8 or 9

11. exp blood transfusion/

12. ((allogenic or allogeneic) adj3 blood transfusion*).ti,ab.

13. (blood component* adj3 transfusion*).ti,ab.

14. ((erythrocyte* or leukocyte* or platelet* or RBC or red blood cell* or WBC or white blood cell* or thrombocyte* or blood) adj3

Transfusion*).ti,ab.

15. 11 or 12 or 13 or 14

16. 10 and 15

17. exp Randomized Controlled Trial/

18. exp controlled clinical trial/

19. placebo.ab.

20. randomi?ed.ti,ab.

21. *Clinical Trial/

22. randomly.ab.

23. trial.ti.

24. 17 or 18 or 19 or 20 or 21 or 22 or 23

25. exp animal/ not (exp human/ and exp animal/)

26. 24 not 25

27. 16 and 26

**CINAHL Plus (EBSCO)**

S1 (MH “Blood Component Removal+”)

S2 (MH “Cytapheresis+”)

S3 TX plasmapheresis or cytapheres* or apheresis or plateletpheresis or pheresis or phereses or aphereses or leukapheresis or leucapheresis

S4 TX Leukoreduc* or leukodeplet* or leukoﬁlt* or leukocyte-reduc* or leucoreduc* or leucodeplet* or leucoﬁlt* or desleucotizat

S5 TX buffy coat-depleted

S6 TX leukocyte count or leukocyte free or leucocyte count or leucocyte free

S7 TX (Blood or white blood cell* or WBC or plasma) N3 (reduc* or deplet* or replete* or remov* or ﬁltrat* or ﬁlter* or cytapheresis)

S8 TX (leukocyte* or leucocyte*) N3 (reduc* or deplet* or replete* or remov* or ﬁltrat* or ﬁlter*)

S9 S1 or S2 or S3 or S4 or S5 or S6 or S7 or S8

S10(MH “Blood Transfusion+”)

S11 TX (allogenic or allogeneic) N3 blood transfusion*

S12 TX blood component* N3 transfusion*

S13 TX (erythrocyte* or leukocyte* or platelet* or RBC or red blood cell* or WBC or white blood cell* or thrombocyte* or blood)

N3 Transfusion*
S14 S10 or S11 or S12 or S13
S15 S9 and S14
S16 (MH “Clinical Trials”)
S17 PT clinical trial*
S18 TX clinical N3 trial*
S19 TI ( (singl* N3 blind*) or (doubl* N3 blind*) or (trebl* N3 blind*) or (tripl* N3 blind*) ) or TI ( (singl* N3 mask*) or (doubl* N3 mask*) or (trebl*N3 mask*) or (tripl*N3 mask*) ) or AB ( (singl*N3 blind*) or (doubl* N3 blind*) or (trebl*N3 blind*) ) or AB ( (singl* N3 mask*) or (doubl* N3 mask*) or (trebl* N3 mask*) or (tripl* N3 mask*) )
S20 TX randomi?ed N3 control* N3 trial*
S21 (MH “Placebos”)
S22 TX placebo*
S23(MH “Random Assignment”)
S24 TX random* N3 allocat*
S25 MH quantitative studies
S26 S16 or S17 or S18 or S19 or S20 or S21 or S22 or S23 or S24 or S25
S27 S15 and S26 Limiters - Exclude MEDLINE records

**LILACS**

((Pt randomized controlled trial OR Pt controlled clinical trial ORMh randomized controlled trials ORMh random allocation ORMh

double-blind method OR Mh single-blind method) AND NOT (Ct animal AND NOT (Ct human and Ct animal)) OR (Pt clinical

trial OR Ex E05.318.760.535$ OR (Tw clin$ AND (Tw trial$ OR Tw ensa$ OR Tw estud$ OR Tw experim$ OR Tw investiga$))

OR ((Tw singl$OR Tw simple$OR Tw doubl$OR Tw doble$OR Tw duplo$OR Tw trebl$OR Tw trip$) AND (Tw blind$OR Tw

cego$OR Tw ciego$ OR Tw mask$OR Tw mascar$)) OR Mh placebos OR Tw placebo$OR (Tw random$ OR Tw randon$ OR Tw

casual$OR Tw acaso$ OR Tw azar OR Tw aleator$) OR Mh research design) ANDNOT (Ct animal ANDNOT (Ct human and Ct

animal)) OR (Ct comparative study OR Ex E05.337$ OR Mh follow-up studies OR Mh prospective studies OR Tw control$OR Tw

prospectiv$ OR Tw volunt$ OR Tw volunteer$) AND NOT (Ct animal AND NOT (Ct human and Ct animal))) AND (Medicina

Transfusional or la transfusión or As transfusões or blood transfusion or Transfusión Sanguínea)

**Clinicaltrials.gov**

( leuk* OR leuc* OR plasmapheresis OR cytapheres OR apheresis ) [DISEASE] AND transfusion [TREATMENT]

**WHO Clinical Trials Registry Platform Search Portal (http://apps.who.int/trialsearch/)**

Condition: leuk* OR leuc* OR plasmapheresis OR cytapheres OR apheresis

Recruitment status: ALL
